# Impact of abrupt sea ice loss on Greenland water isotopes during the last glacial period

**DOI:** 10.1073/pnas.1807261116

**Published:** 2019-02-13

**Authors:** Louise C. Sime, Peter O. Hopcroft, Rachael H. Rhodes

**Affiliations:** ^a^Ice Dynamics and Palaeoclimate, British Antarctic Survey, Cambridge CB3 0ET, United Kingdom;; ^b^School of Geography, Earth and Environmental Sciences, University of Birmingham, Edgbaston B15 2TT, United Kingdom;; ^c^Department of Earth Sciences, University of Cambridge, Cambridge CB2 3EQ, United Kingdom

**Keywords:** abrupt warmings, climate change, Arctic, sea ice, paleoclimate

## Abstract

Greenland ice cores provide excellent evidence of past abrupt climate changes. However, there is no universally accepted theory of how and why these Dansgaard–Oeschger (DO) events occur. Several mechanisms have been proposed to explain DO events, including sea ice, ice shelf buildup, ice sheets, atmospheric circulation, and meltwater changes. DO event temperature reconstructions depend on the stable water isotope ($\delta^{18}$O) and nitrogen isotope measurements from Greenland ice cores: interpretation of these measurements holds the key to understanding the nature of DO events. Here, we demonstrate the primary importance of sea ice as a control on Greenland ice core $\delta^{18}$O: 95% of the variability in $\delta^{18}$O in southern Greenland is explained by DO event sea ice changes. Our suite of DO events, simulated using a general circulation model, accurately captures the amplitude of $\delta^{18}$O enrichment during the abrupt DO event onsets. Simulated geographical variability is broadly consistent with available ice core evidence. We find an hitherto unknown sensitivity of the $\delta^{18}$O paleothermometer to the magnitude of DO event temperature increase: the change in $\delta^{18}$O per Kelvin temperature increase reduces with DO event amplitude. We show that this effect is controlled by precipitation seasonality.

## Significance

The Dansgaard–Oeschger events contained in Greenland ice cores constitute the archetypal record of abrupt climate change. An accurate understanding of these events hinges on interpretation of Greenland records of oxygen and nitrogen isotopes. We present here the important results from a suite of modeled Dansgaard–Oeschger events. These simulations show that the change in oxygen isotope per degree of warming becomes smaller during larger events. Abrupt reductions in sea ice also emerge as a strong control on ice core oxygen isotopes because of the influence on both the moisture source and the regional temperature increase. This work confirms the significance of sea ice for past abrupt warming events.

Sea ice is a key player in the Arctic climate system: it affects precipitation, mass balance, and atmospheric circulation over a large region. Understanding sea ice losses during past abrupt warming events remains challenging (1–7), with the critical relationships between total Arctic (here defined as all Northern Hemisphere) sea ice cover, local climate, and Greenland ice core records still only very poorly understood (8, 9). This is particularly important, since Dansgaard–Oeschger (DO) events are both the largest and best-documented examples of abrupt climate change (10–18).

There has recently been significant progress in reconstructing abrupt DO temperatures increases over Greenland using nitrogen isotopes $\delta^{15}-N_{2}$ (12, 19). This work indicates jumps in temperature over Greenland of up to 16.5 ± 3 K within a few decades (12, 19). A logical but challenging next step is to elucidate how geographical patterns of change in key ice core records, particularly $\delta^{18}$O, from Greenland ice cores can be used to provide that crucial missing information on the nature and cause of abrupt warming events, sea ice loss, and its relationship to abrupt temperature rises (20, 21).

**Fig. 1.**
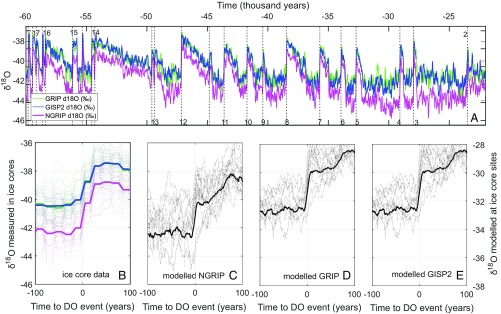
DO events in high-resolution Greenland ice core data and equivalent data from isotope-enabled GCM simulations. All ice cores are on the Greenland Ice Core Chronology 2005 (GICC05) timescale. (*A*) The data from the NGRIP, GRIP, and GISP2 ice cores between 60 and 22 ka from ref. 9, with DO numbers in black. (*B*) All major DO abrupt warming events are shown from −100 to +100 y relative to the identified abrupt warming events (30). Simulated results at the (*C*) NGRIP, (*D*) GRIP, and (*E*) GISP2 ice core sites from the simulations that show significant DO events as identified in *SI Appendix*, Fig. S1. Individual events are depicted with dashed lines; mean values are shown using bold lines. Note that modeled $\delta^{18}$O is on the right axis and that equivalent observed ice core values are on the left axis. The scale is equivalent on both *y* axes, but it is shifted to plot the heavier than observed simulated $\delta^{18}$O values.

DO events are imprinted across the whole of Greenland: wherever last glacial ice is preserved, ice core measurements capture these events (10–12, 19, 22). However, the magnitude of the DO imprint is not identical across the Greenland ice sheet. Early DYE3 ice core measurements suggest that $\delta^{18}$O changes during DO warmings may be larger in the south of Greenland (10) compared with central Greenland. More recent ice core data (Fig. 1 *A* and *B*) imply that, while the magnitudes of DO temperature and accumulation changes (from $\delta^{15}-N_{2}$ and $\delta^{18}$O) are larger in central Greenland compared with the north and northwest (12), $\delta^{18}$O changes could be larger and are likely more variable in the north and northwest compared with central sites (9, 12, 22). How this spatial variability relates to sea ice loss is currently unknown.

General circulation model (GCM) simulations of $\delta^{18}$O enable robust interpretation of records recovered from Greenland ice cores. In particular, they allow us to probe influences on the geographical patterns on the measured $\delta^{18}$O change. The ability to decode DO changes from $\delta^{18}$O records from Greenland ice cores is thus vital to test ideas about drivers of past abrupt climate change (20, 23–25). Here, we present results from a large ensemble of 32 isotope-enabled GCM simulations of DO-type events.

Our DO-type simulations use a freshwater hosing-type setup. Salt is progressively lost from the North Atlantic during stadial periods; classic hosing (26, 27) mechanisms explain the stadial North Atlantic region cooling that we simulate. After a switch of forcing in the hosing, salt returns to the North Atlantic from the tropical Atlantic Ocean and the wider global ocean. This causes the onset of an abrupt warming DO event.

We generate a suite of stadial climates from a 1,500-y glacial period spin-up simulation and then branch 32 different simulations of DO-type warming events off from this range of stadial climates (Fig. 1 *C*–*E* and *SI Appendix*, Fig. S1). The simulations feature different amplitudes of effective salt fluxes alongside the range of initial stadial sea ice states. When calculating stadial–interstadial differences, 50 y of data are used for each climate: the 20 y on either side of the salt flux switch are excluded. These simulations provide a means to unlock the Greenland $\delta^{18}$O records of abrupt DO climate change.

We compare our simulations with high-resolution isotopic records from the last glacial period available from the NGRIP, GRIP, and GISP2 ice cores (11, 28, 29). In addition to the available Greenland ice core $\delta^{18}$O data, a recent identification of DO events is used to locate the abrupt warming transitions for each event (30).

## Comparison with Records of $\delta^{18}$O from Ice Cores

**Fig. 2.**
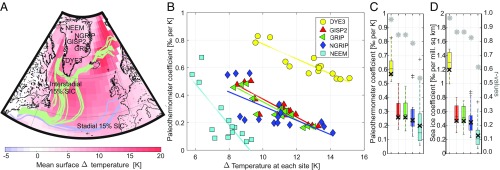
(*A*) Mean surface temperature change from simulated stadial to interstadial climates. Averages are from 15 simulations with significant (+2.0‰) DO rises in $\delta^{18}$O as shown in *SI Appendix*, Fig. S1. Lines show the standard 0.15 mean annual sea ice concentration (SIC) contour for this subset of stadials (blue) and interstadials (green). (*B*) Paleothermometer values for each individual simulated DO warming event. Coefficients are shown from five ice core sites. Each coefficient is calculated for a single DO event (*Materials and Methods*). Lines indicate the tendency of the paleothermometer values to decrease with the size of warming at each site. Larger variability in these paleothermometer values can be seen at NEEM and to a lesser expect, at NGRIP. (*C*) The same as *B* as a boxplot for each ice core site (colors are the same as in *B*). Any outliers are shown as + symbols. There is a clear increase in the coefficients from the north to the south. (*D*) The same as *C* but for sea ice coefficients. Higher coefficients and *r* values suggest that sea ice reconstructions based on DYE3 ice would be invaluable. Note that *r* values (gray stars) and coefficients (bold black crosses) derived from least squares best fits shown in *SI Appendix*, Fig. S2 are plotted for comparison.

**Fig. 3.**
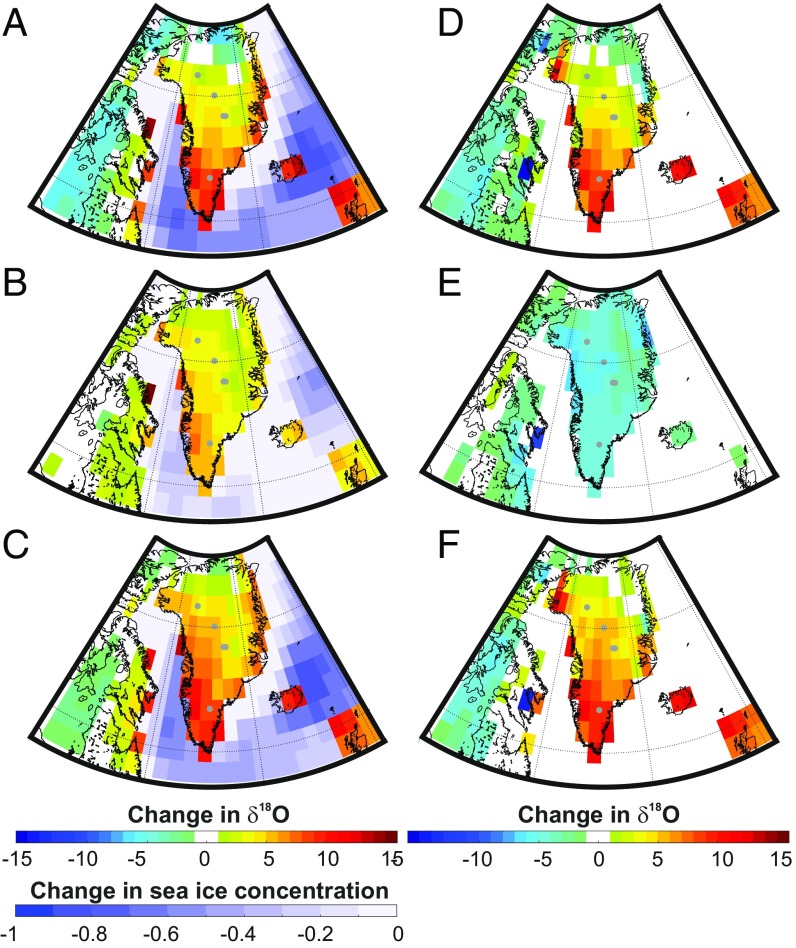
The geographical pattern of changes in $\delta^{18}$O, sea ice, and a decomposition of $\Delta \delta^{18}$O. (*A–C*) Three example simulations illustrate a range of DO sea ice and associated $\delta^{18}$O changes. (*D*) Mean simulated change in stadial and interstadial $\Delta \delta^{18}$O across Greenland for our subset of DO simulations and its decomposition to elucidate the impact of (*E*) changes in precipitation seasonality ($\Delta P_{seas}$) and (*F*) changes due to monthly isotopic composition of precipitation impacts ($\Delta \delta^{18}O_{seas}$). All anomalies (*D* and *E*) are calculated relative to the mean stadial value. Ice core sites are marked with gray dots.

Comparison of simulated DO-type warmings in simulations with significant $\delta^{18}$O jumps with equivalent Greenland ice core measurements (of DO-type abrupt temperature rises) shows good model–data agreement in the magnitude and rate of the abrupt rises in $\delta^{18}$O (Fig. 1 and *SI Appendix*, Fig. S1). Within uncertainties, the magnitudes of modeled precipitation and temperature increases (Table 1) are also in agreement with ice core observations (12, 19). Increases in temperature and $\delta^{18}$O during DO events are always largest in the far south, with smaller changes in the north (Figs. 2*A* and 3). However, the modeled elevations are too low in the south (*Materials and Methods*). This likely contributes to the larger $\delta^{18}$O response at DYE3. A small region in northeast Greenland shows negative $\delta^{18}$O changes in some but not all simulations (Fig. 3).

**Table 1.** Change from simulated stadial to interstadial climates at the sites of Greenland ice cores

Core site	$\delta^{18}$O (‰)	Temp (K)	Precipitation (mm a^−1^)	Precipitation (%)	$\Delta P_{seas}$ (‰)	$\Delta \delta^{18}O_{seas}$ (‰)
NEEM	2.1 ± 1.3	9.1 ± 1.3	12 ± 2	58 ± 11	−5.6 ± 0.82	4.2 ± 1.8
NGRIP	3.2 ± 0.9	11 ± 1.7	21 ± 5.5	77 ± 22	−5.8 ± 1.2	5.7 ± 2
GISP2	3.3 ± 0.7	10 ± 1.6	38 ± 9.8	82 ± 24	−4.4 ± 1.1	5.4 ± 1.7
GRIP	3.1 ± 0.7	10 ± 1.5	37 ± 10	86 ± 26	−4.3 ± 1.1	5.2 ± 1.7
DYE3	9.3 ± 0.5	11 ± 1.6	340 ± 60	120 ± 26	−5.5 ± 0.72	12 ± 1.2

Averages are from 15 simulations with significant (+2.0‰) DO rises in δ^18^O as shown in *SI Appendix*, Fig. S1. Uncertainties presented are ±1 SD from within that set of simulations.

Much of the near-Greenland sea ice loss tends to occur in the southwest on the east side of the Labrador Sea (Fig. 2*A*). The geographical variability in $\delta^{18}$O individual simulations corresponds to the individual pattern of near-Greenland sea ice loss (Fig. 3 *A*–*C*). A similar geographical pattern also occurs in the temperature change patterns recorded in ice cores (12, 20),and in modeled stadial–interstadial temperature changes (15), lending credence to these simulations. The $\delta^{18}$O increase is always strongly positive in the southwest of Greenland, including at DYE3, most commonly with a gradual reduction toward zero change in the northeast. However, some simulated DO events exhibit a stronger east–west geographical gradient in $\delta^{18}$O change (Fig. 3*B*), and others exhibit a stronger south–north gradient (Fig. 3*C*). Thus, although all simulations have large $\delta^{18}$O increases at the most southern DYE3 ice core site, $\delta^{18}$O increases are more variable between simulations at the central and northern sites (Fig. 3 *A*–*C*). During some simulated events, NEEM changes are larger than those at GISP2 and GRIP and of the same magnitude of those at NGRIP.

## Greenland Ice Core Paleothermometers

The $\delta^{18}$O–temperature, or traditional paleothermometer (31), coefficient ($\delta^{18}$O per Kelvin) over the DO warmings varies considerably across Greenland (Fig. 2 *B*–*D*). To reconstruct DO temperature changes from $\delta^{15}-N_{2}$, a model of firn densification and heat diffusion into the ice is required. This uses initial estimates of accumulation rate, ice age, and temperature, where the latter is derived from $\delta^{18}$O. Better independent constraints on accumulation rate and temperature from $\delta^{18}$O can thus reduce uncertainties on the reconstruction of abrupt DO temperature and accumulation change from $\delta^{15}-N_{2}$ (12, 19).

It has previously been proposed that the $\delta^{18}$O–temperature relationship is dependent on obliquity insolation forcing (19). Here, we investigate another possibility. We calculate the paleothermometer coefficient associated with each individual simulated DO event (i.e., using coefficients from $\Delta \delta^{18}$O and Δ temperature) at each ice core site. With this approach applied to 15 simulations with significant (+2.0‰) DO rises in $\delta^{18}$O (*SI Appendix*, Fig. S1), we obtain a mean paleothermometer coefficient of 0.63 at DYE3; GISP2 yields 0.31. At GRIP, it is 0.30. NGRIP is 0.29, and at NEEM, it is 0.23‰ per Kelvin. A very similar geographical pattern emerges regardless the type of approach used to calculate the paleothermometer, with high values in the south and low values in the north. Within uncertainties, the simulated coefficients match the few observed coefficients (*Materials and Methods*) (12, 19). Results here support the idea of considerable variability between DO events in the $\delta^{18}$O–temperature relationship (12, 19).

Our simulations allow us to decipher what controls $\delta^{18}$O–temperature variability between DO events and between ice core sites. In our ensemble, the magnitude of the DO temperature increase has a strong control over the paleothermometer coefficient. Lower $\delta^{18}$O–temperature coefficients occur during larger DO events (Fig. 2*B*) (i.e., we find a strong systematic relationship between the size of the abrupt warming and the paleothermometer coefficient at all Greenland ice core sites). This finding also provides support for the idea that the paleothermometer is fundamentally dependent on the change in temperature at high latitude (32). The pattern varies over Greenland: small decreases occur in the paleothermometer coefficient with warming event size at DYE3 compared with large decreases in the coefficient at NEEM. This identification of a systematic dependence of the $\delta^{18}$O–temperature relationship on the size of the abrupt warming may be useful in further constraining Greenland abrupt temperature change records based on $\delta^{15}-N_{2}$ and $\delta^{18}$O measurements.

## Precipitation and $\delta^{18}$O Seasonality Changes

The current prevailing hypothesis is that most Greenland geographical variability in $\Delta \delta^{18}$O is due to geographical variability in the seasonality of precipitation (9, 12, 19). To test this and to better understand the sea ice imprint of DO and the $\delta^{18}$O–temperature relationship, we calculate the impact of $\delta^{18}$O by only archiving climate information during periods of snow accumulation (33).

If a higher fraction of the yearly precipitation falls during winter months, the $\delta^{18}$O record will have a negative $\delta^{18}$O bias. In addition to this, the average $\delta^{18}$O in each month will also change as the climate moves from a stadial to an interstadial state. We quantify these two effects (by isolating the impact of changes in $\delta^{18}$O due to changes in the seasonal cycle of precipitation) and seasonal changes in $\delta^{18}$O (34). This is a quantification of local precipitation seasonality $\Delta P_{seas}$ vs. other $\Delta \delta^{18}O_{seas}$ impacts on changes in $\delta^{18}$O ($\Delta \delta^{18}$O) (*Materials and Methods*).

The seasonal cycle of precipitation and $\delta^{18}$O both change during a DO event; a larger proportion of precipitation falls during colder months under the warmer interstadial climate relative to the cooler stadial climate. While these changes are less important in driving the majority of the geographical variability (or intercore differences) in $\delta^{18}$O across Greenland, compared with the pattern of near-Greenland sea ice loss, they are critical for understanding why sea ice controls on $\Delta \delta^{18}$O vary so strongly across Greenland. In particular, the huge decreases in paleothermometer coefficients during larger warming events are dependent on changes in seasonality. Intensifications of precipitation seasonality under larger DO events reduce $\Delta \delta^{18}$O everywhere in Greenland, but due to smaller values of $\Delta \delta^{18}O_{seas}$ in the north, the impact of precipitation seasonality is key in this region (Figs. 2 *C* and *D* and 3).

Intensifications of wintertime precipitation due to a larger wintertime area of open water around Greenland occur between stadials and interstadials (*SI Appendix*, Fig. S5). This registers as negative $\Delta P_{seas}$ (Fig. 3*E* and Table 1). Average ice core $\Delta P_{seas}$ is between −4.3 and −5.8‰, which exceeds the size of the recorded DO $\delta^{18}$O rise for four of five ice core sites.

Countering this, $\Delta \delta^{18}O_{seas}$ is considerably enriched, with an increase of between +4.2 and +12‰ across the ice core sites (Fig. 3*F* and Table 1). The change in evaporation in the ensemble is linearly dependent on sea ice coverage (*SI Appendix*, Fig. S6), with a strong dependence on the location where sea ice is reduced (*SI Appendix*, Fig. S7). This increase in (local) evaporation provides explanation for why accumulation and $\delta^{18}$O tend to rise in the vicinity of sea ice loss. As sea ice retreats during the interstadial, evaporation occurs much closer to Greenland (*SI Appendix*, Fig. S7); this moisture travels a much shorter distance to Greenland and therefore, is much less depleted than the moisture arriving over Greenland during the stadial when the sea ice edge is up to 20° farther south.

$\Delta P_{seas}$ is rather uniform across Greenland, but the geographical pattern of $\Delta \delta^{18}O_{seas}$ exerts considerable influence on how sea ice and temperature changes are recorded across Greenland. Thus, contrary to the reasonable current hypothesis that most Greenland geographical variability in $\Delta \delta^{18}$O is due to geographical variability in the seasonality of precipitation (9, 12, 19), instead it is $\Delta \delta^{18}O_{seas}$, which is much more important than $\Delta P_{seas}$ in driving the majority of geographical variability in $\Delta \delta^{18}$O across Greenland.

While the huge increase in $\Delta \delta^{18}O_{seas}$ at the ice core sites more than compensates for negative $\Delta P_{seas}$ in the south, there is a fine balance in the central and northern regions between $\Delta P_{seas}$ and $\Delta \delta^{18}O_{seas}$. This is due to the weak $\Delta \delta^{18}O_{seas}$ imprint. This results in weak relationships between $\delta^{18}$O and sea ice and the associated $\delta^{18}$O–temperature paleothermometer relationship (Fig. 2 *B* and *C*). Understanding these two large opposing changes, with distinctly different geographical patterns, is key to understanding Greenland DO $\delta^{18}$O changes.

## Role of Sea Ice

In the north of Greenland, there is an extraordinary simulated range of DO event behaviors. This can be seen in the NEEM $\delta^{18}$O changes and the $\delta^{18}$O–temperature relationships at NEEM and partly, NGRIP (Fig. 2 and Table 1): NEEM shows the largest range of $\delta^{18}$O–temperature coefficients (Fig. 2*C*), compared with any of the other ice cores sites. The amount of variance in $\delta^{18}$O for the largest simulated DO events directly explained by temperature (sea ice changes) is less than 29% (39%) at NEEM. However, at the other end of Greenland, the record of $\delta^{18}$O change at DYE3 for the largest events is nearly entirely 95% explained by sea ice changes (Fig. 2 *C* and *D*, *r* values converted to explained variances).

Although it is not possible to unambiguously attribute $\delta^{18}$O changes to particular components, like sea ice, temperature, atmospheric circulation, or storm tracks, the similar patterns of $\delta^{18}$O–temperature and $\delta^{18}$O–sea ice relationships (Fig. 2 *C* and *D*), higher explained variances for sea ice over temperature (95% for sea ice vs. 92% for temperature at DYE3 and 70 vs. 62% at NGRIP), and sea ice impacts on the moisture sources and transport to Greenland suggest that sea ice exerts an even greater control on the stadial–interstadial $\delta^{18}$O over Greenland than temperature. This is because sea ice change controls both temperature in the wider region and the moisture availability.

This demonstration of the importance of the sea ice imprint on DO event Greenland $\Delta \delta^{18}$O should help open the door to quantitative reconstructions of abrupt DO sea ice change based on these ice core measurements. Finally, these results show that precise and well-dated measurements of $\delta^{18}$O from DYE3 or from other new southern dome cores alongside careful isotopic-enabled modeling (35, 36) would be invaluable in quantifying changes in Arctic Sea ice during DO events.

## Materials and Methods

### Model Simulations.

Global modeling studies with intermediate complexity models have shown bistability of the Atlantic Meridional Overturning Circulation (AMOC) in response to oscillatory or stochastic freshwater forcing in the North Atlantic (26). However, this behavior does not always appear in fully coupled 3D GCMs (18, 37, 38). Recently, however, one such model (Community Earth System Model 1) has been shown to predict significant nonforced oscillations in the strength of the AMOC and North Atlantic temperatures when subject to last glacial maximum (LGM) boundary conditions (27). This behavior has been termed a “kicked” salt oscillator. At the start of a DO-type warming, a large northward North Atlantic flux of salt occurs. This is associated with abruptly increased northward delivery of ocean heat and the melt of substantial areas of North Atlantic sea ice (5, 6). A gradual external forcing related to orographic change of the North American Laurentide ice sheet or a small freshwater perturbation can also trigger similar oscillations (4, 15). A version of the GCM called GFDL CM2Mc also exhibits nonforced oscillations of the AMOC, although only for a certain combination of background climate conditions (39). Here, we use a forced salt (or hosing) oscillation approach for setting up our suite of DO-type simulations.

Simulations are set up using an isotope-enabled version of the Hadley Center Coupled Model general circulation model (HadCM3). This GCM consists of a coupled atmosphere, ocean, and sea ice model and has been widely used to study past, present, and future climates (40, 41). HadCM3 can also be run for hundreds of years on modern supercomputers (24, 42, 43). The ocean component of HadCM3 is a rigid lid model, with a fixed volume and water conservation through a time-invariant surface salinity flux that represents iceberg calving. Ref. 44 has the implementation of water isotope code in HadCM3. Ice sheets and sea ice in the model are initialized with $\delta^{18}$O values of −40 and −2‰, respectively. Using this model, salinity fluxes are applied in opposing directions to the North Atlantic vs. the rest of the global ocean surface. Simulations are set up using LGM ice sheets, orbital forcing, and greenhouse gas composition; additional details are in ref. 42. Every DO simulation is continued from the same standard 1,000-y glacial period (LGM) spin-up simulation. In the first instance, each DO-type simulation is then run for 500 y using an identical stadial-type forcing: a negative salinity flux, equivalent to 0.25 Sv of freshwater, is applied across the North Atlantic between 50^○^ N and 70^○^ N. To balance the global salt (freshwater) budget, a coincident equivalent-sized positive salinity flux is also applied to the rest of the global ocean, ensuring a net global salt flux of zero. This generates a 1,500-y spin up of a stadial-type climate.

A suite of DO simulations is branched from this initial spin-up 1,500 stadial-type simulations. To generate the suite of stadial-type climates, a range of salt fluxes is then applied to the 1,500 spin ups from the equivalent of 0.1- to 1.0-Sv North Atlantic freshwater. The salt flux increments are equivalent to freshwater fluxes of 0.1, 0.25, 0.5, and 1.0 Sv (*SI Appendix*, Fig. S1). Later interstadial negative salinity fluxes are applied using the same range of magnitudes. Each of these stadial-type simulations is branched off from a different year of the spin-up simulation. For each of the salt flux experiments, the surface layer of the ocean is freshened across the North Atlantic at a rate equivalent to the addition of between 0.1 and 1 Sv of freshwater, while the rest of the surface layer of the ocean is salinified at a rate equivalent to the loss of between 0.1 and 1 Sv (i.e., from −0.1 to −1 Sv) of freshwater. The suite of simulations is run using this stadial-type forcing for between 100 and 500 y, yielding a wide range of stadial climates. In each case, the North Atlantic (negative) salt forcing is always balanced by an equivalent-sized (positive) salt forcing applied across the rest of the global ocean; therefore, the net global freshwater (or salt) flux is always zero.

To generate a switch between stadial-type and interstadial-type climate states, a reversed salinity forcing, with positive salinity forcing over the North Atlantic and negative values over the remaining global ocean, is then applied. All forcings are between ±0.1 and ±1 Sv. The net global salinity flux is always zero. When we calculate stadial–interstadial difference, we use 50 y of data in each case: the 20 y on either side of the salt flux switch are excluded.

Greenland is represented by 80 grid points in HadCM3; thus, some smoothing of the surface topography is required to run the simulation (43). The modeled surface elevation is thus lower than the observed elevation. For the northern ice core sites, our modeled surface elevations are generally within 500 m of the present day surface elevations: NEEM: 2,450 vs. 2,341 m above sea level (observed vs. modeled, respectively). Similarly, NGRIP is 2,917 vs. 2,788 m above sea level (observed vs. modeled, respectively), and GISP2 is 3,216 vs. 2,832 m above sea level (observed vs. modeled, respectively). Because Greenland is somewhat narrower in the south, the elevations at DYE3 are 2,480 vs. 1,240 m for observed vs. modeled surface elevation, respectively. Thus, while all modeled sites are somewhat too low, the discrepancy is most significant in the south at DYE3. This may account for some, but not all, of the larger sensitivity of $\delta^{18}$O at DYE3 during DO events.

The suite of simulations shows a range of $\delta^{18}$O values and sea ice states (Fig. 2*A* and *SI Appendix*, Fig. S1). At NGRIP, the stadial climate $\delta^{18}$O varies from −37.5 to −29‰. Of 32 initial simulations, 15 exhibit DO-type abrupt warming over Greenland with $\delta^{18}$O increases of 2.0‰ or more, where a 2.0‰ threshold could be considered representative of the minimum for a small DO-type abrupt warming (45). Each simulation with a significant $\delta^{18}$O jump starts from $\delta^{18}$O values at NGRIP of −32‰ or below (*SI Appendix*, Fig. S1). Most of the larger $\delta^{18}$O jumps occur under an interstadial-type salt flux (i.e., a return of salt to the North Atlantic) of size equivalent to 0.25 Sv or larger. This magnitude of salt oscillation is also in agreement with the size of salt flux required to induce significant AMOC variations in HadCM3 under present and future forcing scenarios (37). Note that there is a mean offset in Greenland between the mean model and mean data in $\delta^{18}$O of +8‰ (Fig. 1 *C*–*E*). This offset is in the same direction as in earlier isotopic simulations alongside a similar warm model bias in the temperature and a wet bias in the precipitation (21).

### Temperature and Sea Ice Coefficients.

There are two main approaches that are used to calculate $\delta^{18}$O–temperature coefficients and similarly, $\delta^{18}$O–sea ice coefficients. Note that the terms gradients and slopes are also used to denote these coefficient values. The first and most common approach to calculating the coefficients is to fit a second-degree polynomial to a set of stadial and interstadial $\delta^{18}$O and temperature values, minimizing the least squares term) (46, 47). Coefficients (or gradients or slopes) from this method are shown in Fig. 2 *C* and *D* (bold black crosses) and *SI Appendix*, Figs. S2 and S3.

The second approach is to calculate the coefficients for each individual simulated DO event so that the coefficient in this case is simply $\Delta \delta^{18}$O/Δ temperature for the paleothermometer. Additionally, $\Delta \delta^{18}$O/Δ Arctic sea ice area indicates the sea ice coefficient. Note that Arctic sea ice area is a total Northern Hemisphere value that is calculated by summing each grid box multiplied by the sea ice concentration and grid box area. This approach yields a distribution of coefficients and is arguably a more accurate depiction of the relationship between $\Delta \delta^{18}$O, Δ temperature, and Δ Arctic sea ice area for the suite of simulated DO events. This approach is used to characterize the distribution of paleothermometer and sea ice coefficients (Fig. 2 *B*–*D*, colored and boxplot results).

Using the second approach and the same set of the largest DO events, we obtain a mean paleothermometer coefficient of 0.63‰ per Kelvin (16th to 84th percentile is 0.53 to 0.77) at DYE3; GISP2 yields 0.31‰ per Kelvin (0.20 to 0.46), GRIP is 0.30‰ per Kelvin (0.19 to 0.45), NGRIP is 0.29‰ per Kelvin (0.19 to 0.52), and NEEM is 0.23‰ per Kelvin (0.08 to 0.52). If the first approach is used, a similar geographical pattern and similar values emerge (Fig. 2*C*). This same pattern emerges independent of whether all or subsets of simulations are used in the calculations, although eliminating the smaller DO events does tend to raise the average values of coefficients. While the average simulated NGRIP coefficient is lower than the overall coefficient of 0.52‰ per Kelvin suggested for NGRIP (19), other measurements imply a somewhat lower coefficient (12), and our set of simulations indicates that the observed coefficient of 0.52 at NGRIP occurs around the 84th percentile value (Fig. 2*C*) (i.e., within the central range of values of simulated coefficients). Our range also encompasses the interevent set of approximate initial NGRIP coefficients from 0.28 to 0.42‰ per Kelvin used by ref. 19.

### Uncertainties on Table Values.

The data in Table 1 are mean values from the subset of simulations that have significant (2.0‰) DO increases in $\delta^{18}$O at NGRIP. The uncertainties are ±1 SD calculated from this sample size of 15 DO events.

### Isolating the Impact of Changes in Seasonality.

To qualify the relative impact of the precipitation vs. $\delta^{18}$O seasonal changes, we isolate the impact of changes in $\delta^{18}$O due to changes in the seasonal cycle of precipitation and seasonal changes in $\delta^{18}$O (34) (Fig. 3 *D*–*F* and *SI Appendix*, Fig. S4):

$$\Delta P_{seas}=\frac{\sum_{j}\delta^{18}O_{j}^{stadial}.P_{j}}{\sum_{j}P_{j}}-\frac{\sum_{j}\delta^{18}O_{j}^{stadial}.P_{j}^{stadial}}{\sum_{j}P_{j}^{stadial}}.$$ [1]

Changes in $\delta^{18}$O due solely to monthly changes in $\delta^{18}$O (nonprecipitation seasonality influences) are calculated:

$$\Delta \delta^{18}O_{seas}=\frac{\sum_{j}\delta^{18}O_{j}.P_{j}^{stadial}}{\sum_{j}P_{j}^{stadial}}-\frac{\sum_{j}\delta^{18}O_{j}^{stadial}.P_{j}^{stadial}}{\sum_{j}P_{j}^{stadial}},$$ [2]

where the summations are over 12 mo (index *j*). The superscript *stadial* indicates values from the cool preceding stadial-type simulated climate, and no superscript indicates values from the postwarming interstadial-type climate. This method is somewhat different from that used to decompose $\delta^{18}$O changes in ref. 48, where daily outputs were used. In particular, residuals [i.e., $R_{esidual}$ = $\left(\Delta P_{seas}\;+\;\Delta \delta^{18}O_{seas}\right)\;-\;\Delta \delta^{18}$O] could indicate changes in the higher (than monthly)-frequency covariance between precipitation and $\delta^{18}$O not captured by this monthly “seasonal”-type decomposition. However, the $R_{esidual}$ is smaller than ±2‰ over Greenland (less than ±0.5‰ over most of central Greenland). The stadial to interstadial values are calculated using 50 y of data from each climate, with the 20 y on either side of the abrupt warming excluded from the analysis.

## Supplementary Material

Supplementary File

## References

[1] Gildor H, Tziperman E (2003). Sea-ice switches and abrupt climate change. Philos Trans R Soc Lond A Math Phys Eng Sci.

[2] Li C, Battisti DS, Schrag DP, Tziperman E (2005). Abrupt climate shifts in Greenland due to displacements of the sea ice edge. Geophys Res Lett.

[3] Li C, Battisti DS, Bitz CM (2010). Can North Atlantic sea ice anomalies account for Dansgaard-Oeschger climate signals?. J Clim.

[4] Zhang X, Prange M, Merkel U, Schulz M (2014). Instability of the Atlantic overturning circulation during marine isotope stage 3. Geophys Res Lett.

[5] Vettoretti G, Peltier WR (2016). Thermohaline instability and the formation of glacial North Atlantic super polynyas at the onset of Dansgaard-Oeschger warming events. Geophys Res Lett.

[6] Vettoretti G, Peltier WR (2018). Fast physics and slow physics in the nonlinear Dansgaard–Oeschger relaxation oscillation. J Clim.

[7] Dokken TM, Nisancioglu KH, Li C, Battisti DS, Kissel C (2013). Dansgaard-Oeschger cycles: Interactions between ocean and sea ice intrinsic to the nordic seas. Paleoceanography.

[8] Flückiger J, Knutti R, White J, Renssen H (2008). Modeled seasonality of glacial abrupt climate events. Clim Dyn.

[9] Seierstad IK (2014). Consistently dated records from the Greenland grip, gisp2 and ngrip ice cores for the past 104 ka reveal regional millennial-scale δ18o gradients with possible heinrich event imprint. Quat Sci Rev.

[10] Johnsen SJ (1992). Irregular glacial interstadials recorded in a new Greenland ice core. Nature.

[11] NGRIP Project Members (2004). High-resolution record of northern hemisphere climate extending into the last interglacial period. Nature.

[12] Guillevic M (2013). Spatial gradients of temperature, accumulation and δo-ice in Greenland over a series of dansgaard-oeschger events. Clim Past.

[13] Clement AC, Peterson LC (2008). Mechanisms of abrupt climate change of the last glacial period. Rev Geophys.

[14] Petersen SV, Schrag DP, Clark PU (2013). A new mechanism for Dansgaard-Oeschger cycles. Paleoceanography.

[15] Zhang X, Lohmann G, Knorr G, Purcell C (2014). Abrupt glacial climate shifts controlled by ice sheet changes. Nature.

[16] Seager R, Battisti DS, Lorenz EN, Schneider T, Sobel AH (2007). Challenges to our understanding of the general circulation: Abrupt climate change. The Global Circulation of the Atmosphere.

[17] Knutti R, Fluckiger J, Stocker TF, Timmermann A (2004). Strong hemispheric coupling of glacial climate through freshwater discharge and ocean circulation. Nature.

[18] Liu Z (2009). Transient simulation of last deglaciation with a new mechanism for Bølling-Allerød warming. Science.

[19] Kindler P (2014). Temperature reconstruction from 10 to 120 kyr b2k from the ngrip ice core. Clim Past.

[20] Buizert C (2014). Greenland temperature response to climate forcing during the last deglaciation. Science.

[21] Sime LC (2013). Warm climate isotopic simulations: What do we learn about interglacial signals in Greenland ice cores?. Quat Sci Rev.

[22] Schüpbach S (2018). Greenland records of aerosol source and atmospheric lifetime changes from the eemian to the holocene. Nat Commun.

[23] Sime LC, Wolff EW, Oliver KIC, Tindall JC (2009). Evidence for warmer interglacials in East Antarctic ice cores. Nature.

[24] Holloway MD, Sime LC, Singarayer JS, Tindall JC, Valdes PJ (2016). Antarctic last interglacial isotope peak in response to sea ice retreat not ice sheet collapse. Nat Commun.

[25] Malmierca Vallet I (2018). Simulating the last interglacial Greenland stable water isotope peak: The role of Arctic sea ice changes. Quat Sci Rev.

[26] Ganopolski A, Rahmstorf S (2001). Rapid changes of glacial climate simulated in a coupled climate model. Nature.

[27] Peltier WR, Vettoretti G (2014). Dansgaard-Oeschger oscillations predicted in a comprehensive model of glacial climate: A “kicked” salt oscillator in the atlantic. Geophys Res Lett.

[28] Johnsen SJ (1997). The $\delta^{18}$O record along the Greenland ice core project deep ice core and the problem of possible Eemian climatic instability. J Geophys Res.

[29] Grootes PM, Stuiver M (1997). Oxygen 18/16 variability in Greenland snow and ice with 103 to 105-year time resolution. J Geophys Res.

[30] WAIS Divide Project Members (2015). Precise interpolar phasing of abrupt climate change during the last ice age. Nature.

[31] Jouzel J (2003). Magnitude of isotope/temperature scaling for interpretation of central Antarctic ice cores. J Geophys Res.

[32] Guan J (2016). Understanding the temporal slope of the temperature-water isotope relation during the deglaciation using isocam3: The slope equation. J Geophys Res Atmos.

[33] Krinner G, Genthon C, Jouzel J (1997). GCM analysis of local influences on ice core δ signals. Geophys Res Lett.

[34] Liu X, Battisti DS (2015). The influence of orbital forcing of tropical insolation on the climate and isotopic composition of precipitation in South America. J Clim.

[35] LeGrande AN, Schmidt GA (2008). Ensemble, water isotope–enabled, coupled general circulation modeling insights into the 8.2 ka event. Paleoceanography.

[36] Liu Z (2012). Younger dryas cooling and the Greenland climate response to CO2. Proc Natl Acad Sci USA.

[37] Stouffer RJ (2006). Investigating the causes of the response of the thermohaline circulation to past and future climate changes. J Clim.

[38] Valdes P (2011). Built for stability. Nat Geosci.

[39] Brown N, Galbraith ED (2016). Hosed vs. unhosed: Interruptions of the atlantic meridional overturning circulation in a global coupled model, with and without freshwater forcing. Clim Past.

[40] IPCC (2007).

[41] IPCC (2013).

[42] Holloway MD, Sime LC, Singarayer JS, Tindall JC, Valdes PJ (2016). Reconstructing paleosalinity from $\delta^{18}$O: Coupled model simulations of the last glacial maximum, last interglacial and late holocene. Quat Sci Rev.

[43] Valdes PJ (2017). The bridge hadcm3 family of climate models: Hadcm3@bristol v1.0. Geosci. Model Dev Discuss.

[44] Tindall JC, Valdes PJ, Sime LC (2009). Stable water isotopes in HadCM3: Isotopic signature of El Niño Southern oscillation and the tropical amount effect. J Geophys Res.

[45] Capron E (2010). Millennial and sub-millennial scale climatic variations recorded in polar ice cores over the last glacial period. Clim Past.

[46] Jouzel J (1997). Validity of the temperature reconstruction from water isotopes in ice cores. J Geophys Res Oceans.

[47] Jouzel J (2007). Orbital and millennial Antarctic climate variability over the past 800,000 years. Science.

[48] Sime LC, Tindall J, Wolff E, Connolley W, Valdes P (2008). The Antarctic isotopic thermometer during a CO2 forced warming event. J Geophys Res Atmos.

