# Supplementary material for: Impact of abrupt sea ice loss on Greenland water isotopes during the last glacial period
Source: Proc Natl Acad Sci U S A. 2019 Feb 13;116(10):4099–104. doi: 10.1073/pnas.1807261116 (PMC6410777; doi:10.1073/pnas.1807261116)
Supplement: Supplementary File [file pnas.1807261116.sapp.pdf]

Supplementary Figures

The impact of abrupt sea ice loss on Greenland water isotopes during the Last Glacial Period

Figure S1 The full set of DO simulations. δ^{18}O changes against mean stadial δ^{18}O (at NGRIP) for all 32 DO simulations. The grey dashed box contains the subset of simulations which show a stadial to interstadial increase in δ^{18}O which is greater than 2.0 ‰. Coloured dashed lines show best fits for each set of salt fluxes; see Methods for further details.

Figure S2 Relationship between simulated Arctic sea ice area, temperature and δ^{18}O at the four ice core sites (GISP2 and GRIP are treated here as equivalent), using the subset of simulations highlighted in Figure S1. Best fits, using a least squares minimisation, are shown for each set of results. See also Figure 2cd. Colours indicate DO salt fluxes: red is 0.1 Sv; yellow is 0.25 Sv; cyan is 0.5 Sv; and blue is 1 Sv equivalent. Squares represent stadial simulation states, and circles interstadial states. Circles represent the differences between stadials and interstadials. Panel (a) δ^{18}O versus temperature (or paleothermometer), both stadial and inter-stadial climates at DYE3; (b) δ^{18}O versus Arctic sea ice area, both stadial and inter-stadial results at DYE3; (c,d) as above, but for GISP2-GRIP site; (e,f) as above but for NGRIP; (g,h) as above but for NEEM.

Figure S3 The geographical pattern of paleothermometer and sea ice coefficients across Greenland using the best fit approach; the same approach as shown in Figure S2. (a) Temperature and δ^{18}O coefficients and (b) simulated Arctic sea ice area and δ^{18}O coefficients. Shading indicates the coefficient (gradient), the labelled lines indicate the -0.95, -0.8, -0.5, 0, 0.5, 0.8, and 0.95 correlation coefficent (r) contours.

Figure S4 Changes in precipitation seasonality (ΔP_{seas}). Simulation results are averaged into the bottom, middle, and top thirds by DO temperature increases (at NGRIP). Larger temperature increases tend to be accompanied by larger changes in precipitation seasonality. It is this which reduces the paleothermometer values as abrupt temperature rises increase, particularly at NEEM.

Figure S5 Changes in precipitation, pressure, and surface wind velocity patterns. (a) Mean stadial precipitation (shaded); pressure patterns (contours) - note position of low pressure centred in the North Atlantic; and surface (10 M) wind velocities (arrows). (b) As above, but for mean interstadial conditions. (c) Change in precipitation (as a percentage) between the stadial and interstadial conditions, with associated changes in pressure and wind velocities shown by contours and arrows.

Figure S6 Scatter plot of anomalies in sea ice fraction and area-weighted mean sea surface evaporation (mm/day) averaged over the North Atlantic (70W-20E, 40-70N) in the DO events discussed in the main text, and highlighted in Figure S1. This analysis shows that the larger the decrease in the sea ice area, the larger the increase in evaporation.

Figure S7 Annual mean change in evaporation from the sea surface interstadial minus stadial (mm/day). The two contours show the 15% sea ice edge in the interstadial and stadial climate in each case. The percentage change in sea ice area averaged over the North Atlantic (70W-20E, 40-70N) is given above each figure. The simulations shown correspond to those discussed in the main text, and highlighted in Figure S1.

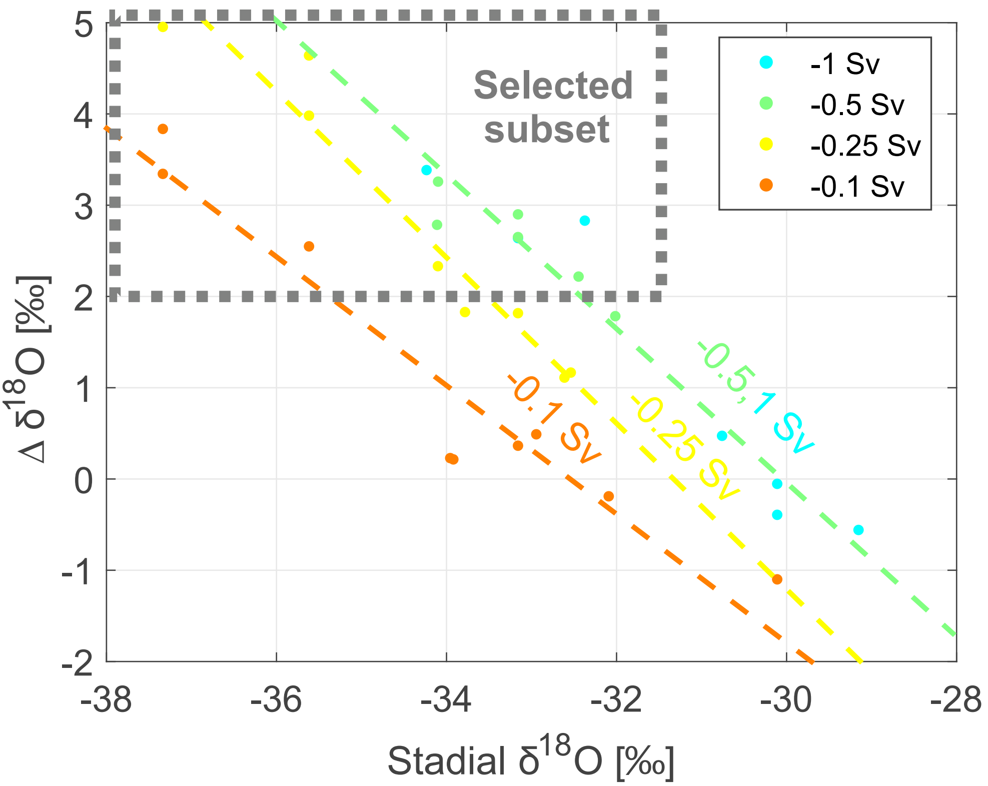

Selected subset
-1 Sv
-0.5 Sv
-0.25 Sv
-0.1 Sv
-0.5, 1 Sv
-0.25 Sv
-0.1 Sv
Δ δ18O [‰]
Stadial δ18O [‰]
5
4
3
2
1
0
-1
-2
-38
-36
-34
-32
-30
-28

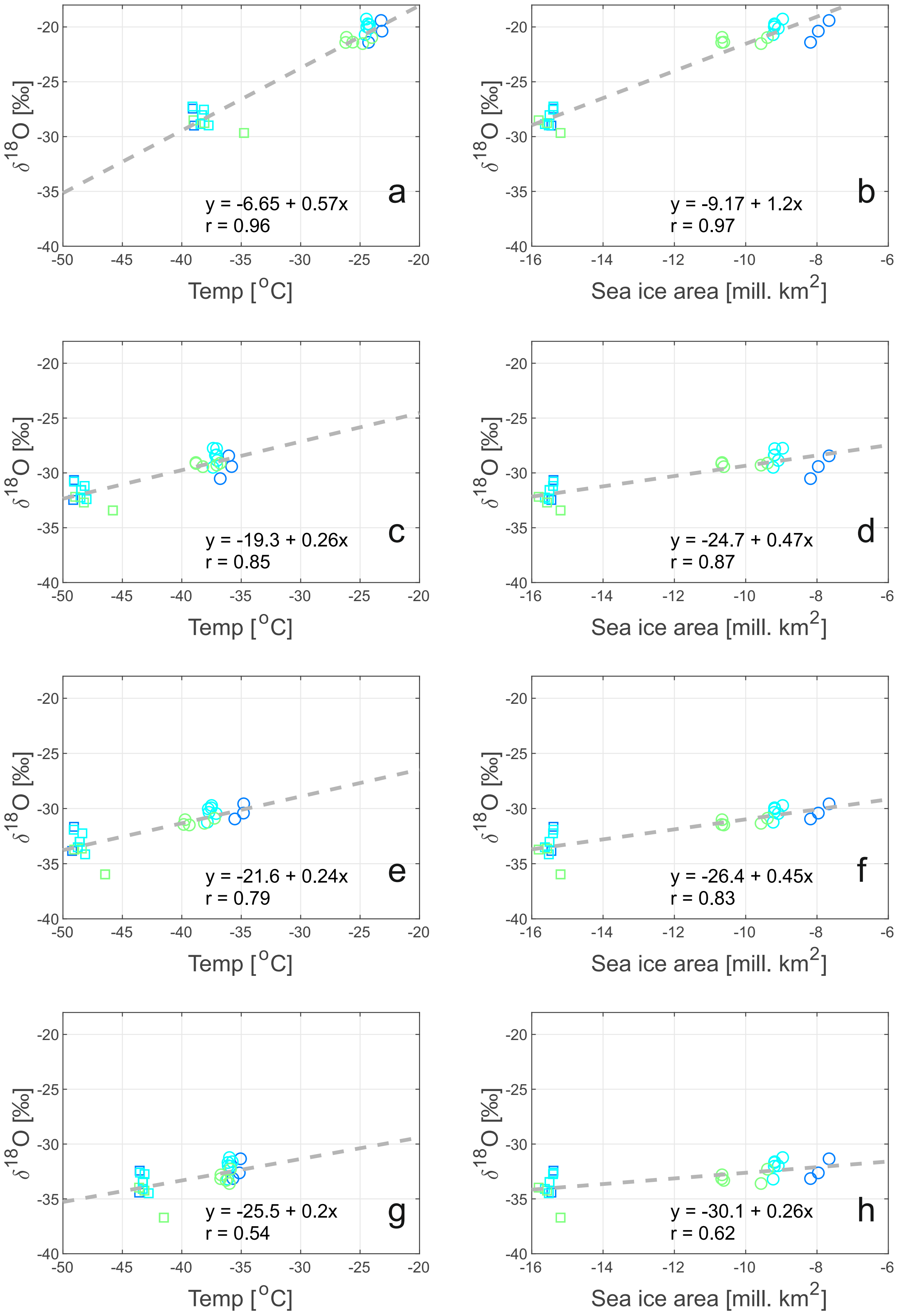

y = -6.65 + 0.57x
r = 0.96
a
y = -9.17 + 1.2x
r = 0.97
b
y = -19.3 + 0.26x
r = 0.85
c
y = -24.7 + 0.47x
r = 0.87
d
y = -21.6 + 0.24x
r = 0.79
e
y = -26.4 + 0.45x
r = 0.83
f
y = -25.5 + 0.2x
r = 0.54
g
y = -30.1 + 0.26x
r = 0.62
h
δ18O [‰]
Temp [°C]
Sea ice area [mill. km2]

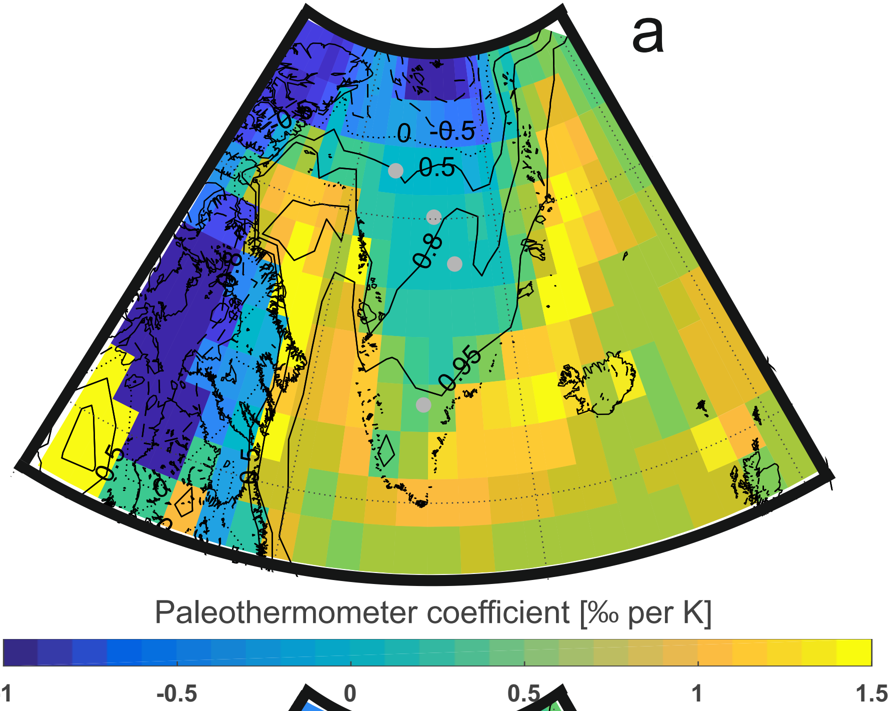
a
0
-0.5
0.5
0.8
0.95
Paleothermometer coefficient [‰ per K]
-1
-0.5
0
0.5
1
1.5

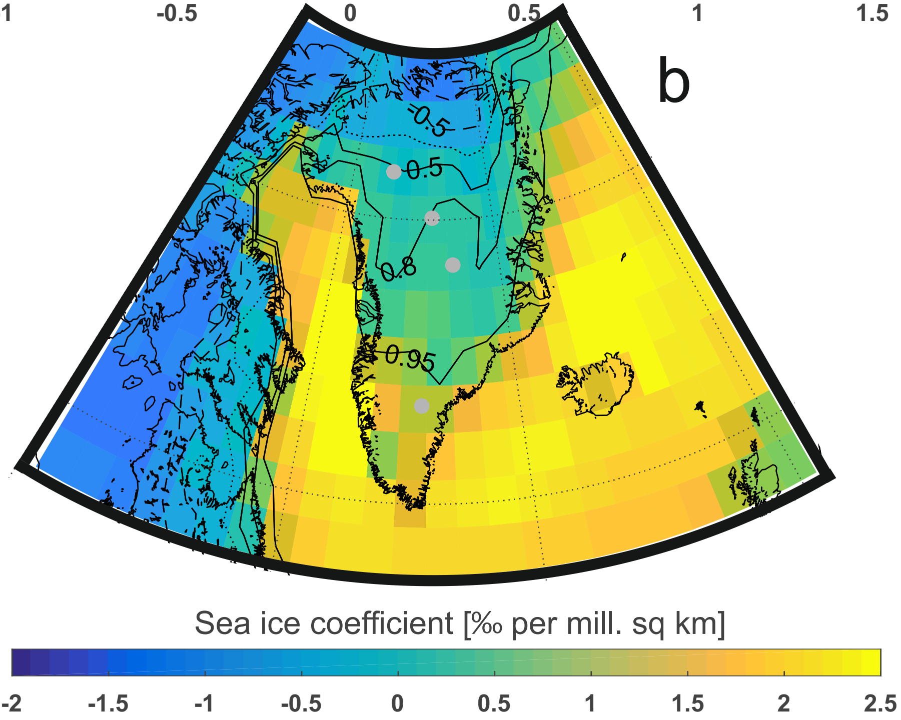
b
-0.5
0.5
0.8
0.95
Sea ice coefficient [‰ per mill. sq km]
-2
-1.5
-1
-0.5
0
0.5
1
1.5
2
2.5

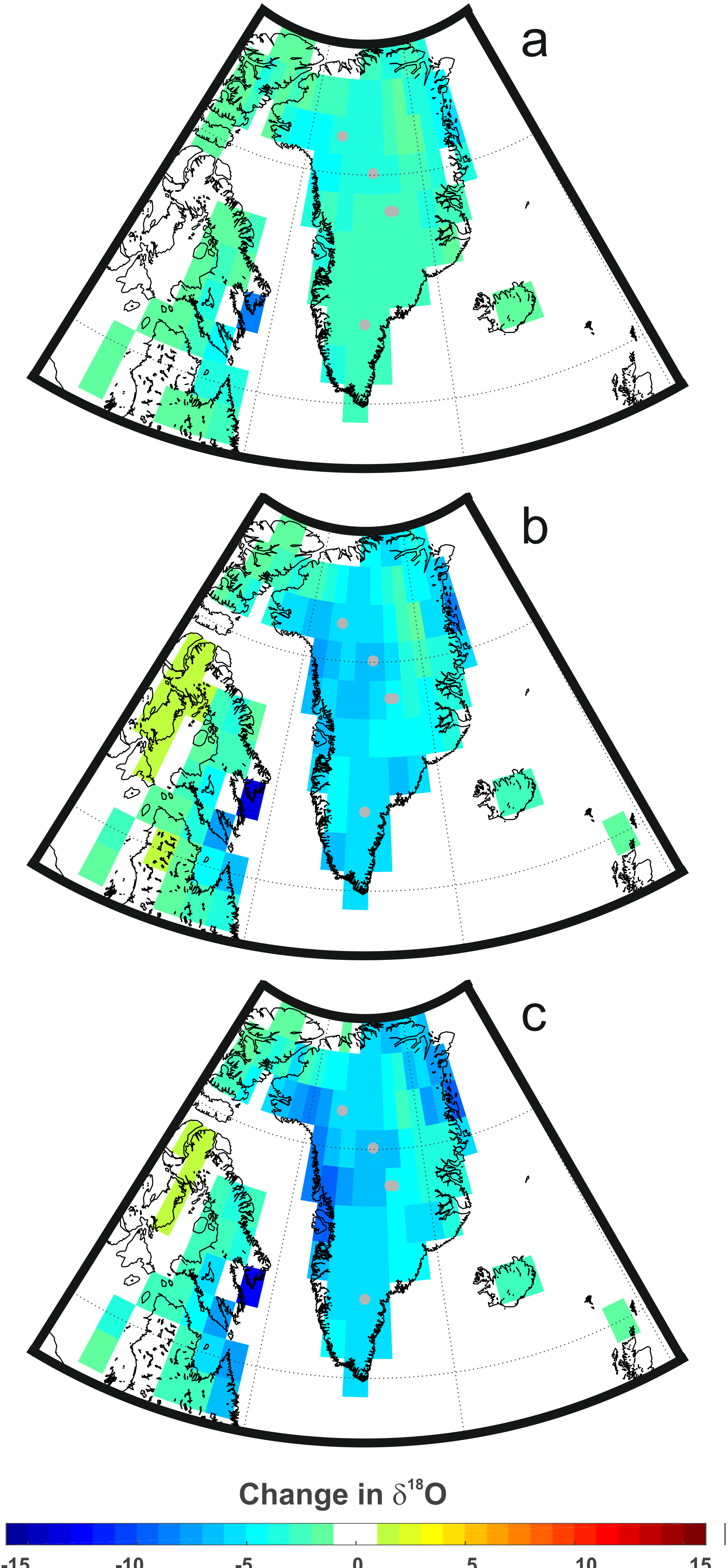
a
b
c
Change in δ18O
-15
-10
-5
0
5
10
15

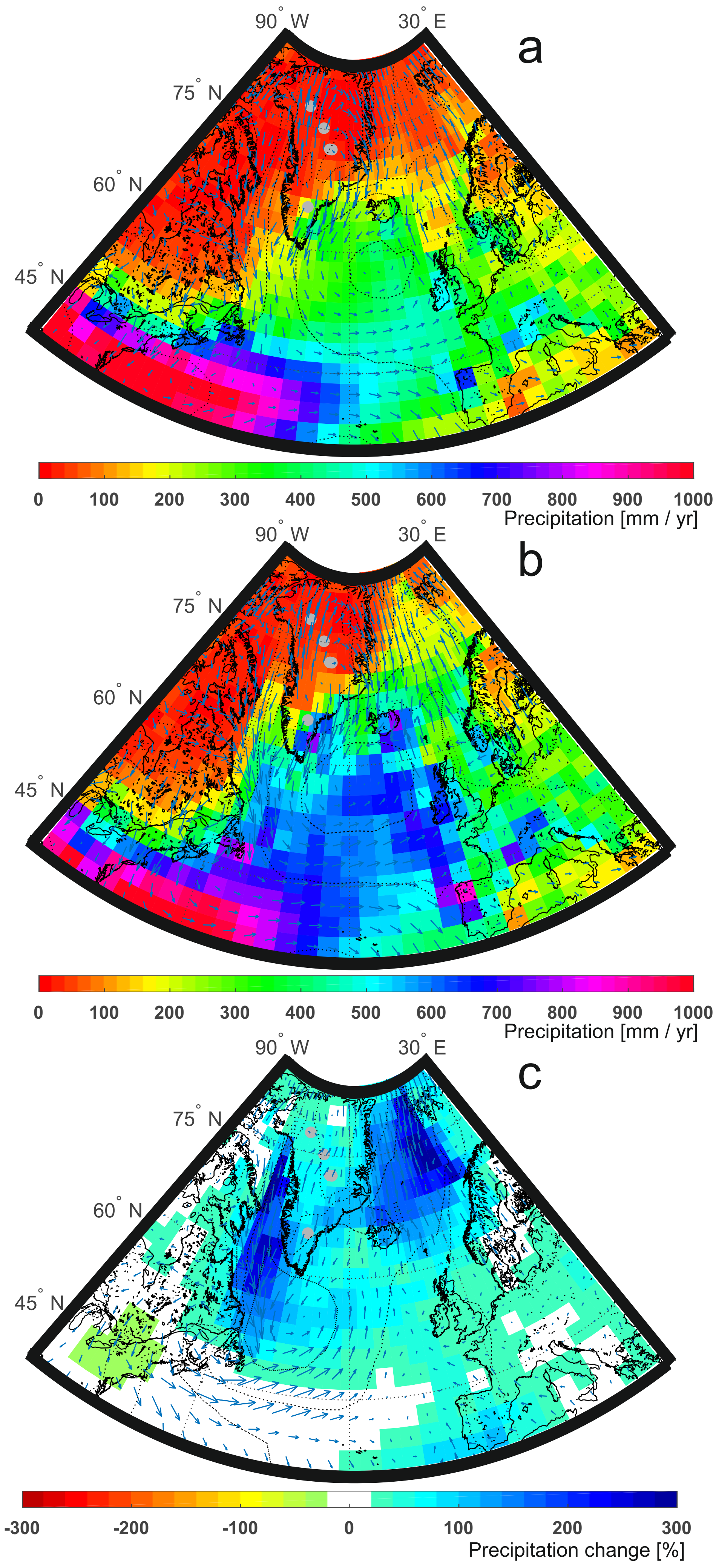
90° W
30° E
a
75° N
60° N
45° N
0
100
200
300
400
500
600
700
800
900
1000
Precipitation [mm / yr]
90° W
30° E
b
75° N
60° N
45° N
0
100
200
300
400
500
600
700
800
900
1000
Precipitation [mm / yr]
90° W
30° E
c
75° N
60° N
45° N
-300
-200
-100
0
100
200
300
Precipitation change [%]

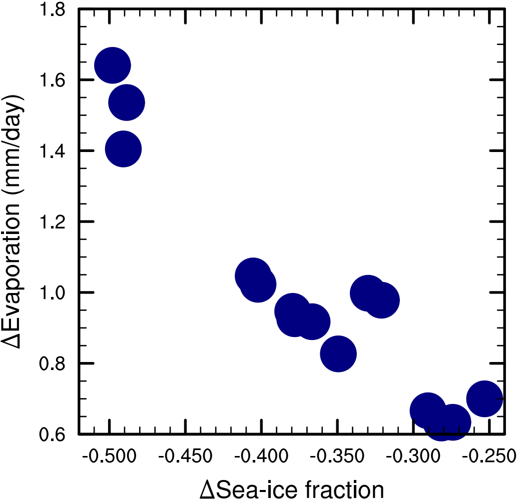
1.8
1.6
1.4
1.2
1.0
0.8
0.6
ΔEvaporation (mm/day)
-0.500
-0.450
-0.400
-0.350
-0.300
-0.250
ΔSea-ice fraction

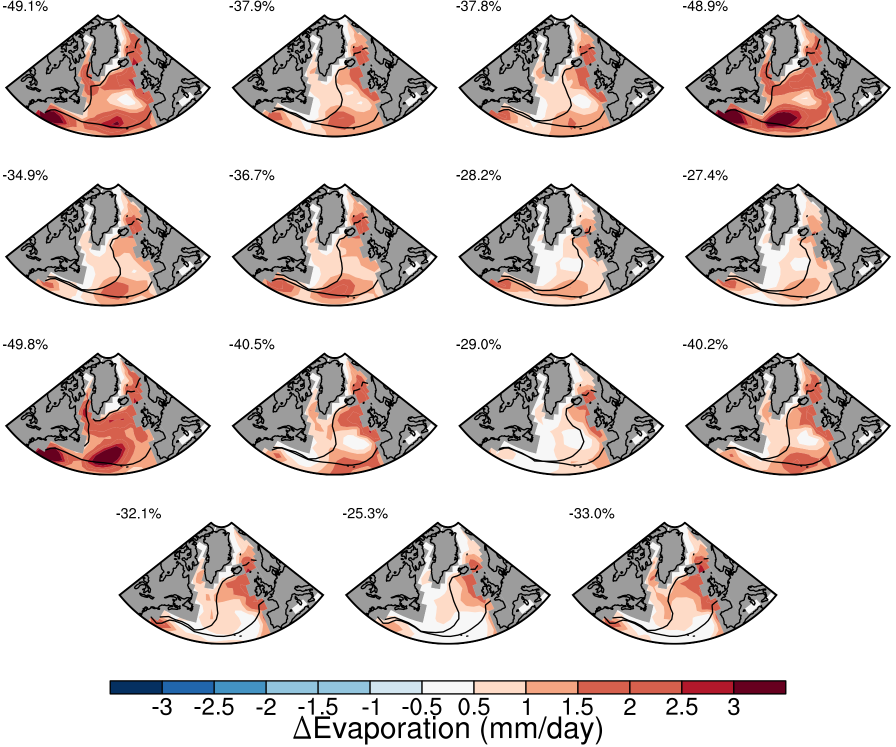
-49.1%
-37.9%
-37.8%
-48.9%
-34.9%
-36.7%
-28.2%
-27.4%
-49.8%
-40.5%
-29.0%
-40.2%
-32.1%
-25.3%
-33.0%
-3
-2.5
-2
-1.5
-1
-0.5
0.5
1
1.5
2
2.5
3
ΔEvaporation (mm/day)
